# Hybrids of cationic porphyrins with nanocarbons

**DOI:** 10.1007/s10847-015-0485-z

**Published:** 2015-03-17

**Authors:** Beata Girek, Wanda Sliwa

**Affiliations:** Institute of Chemistry, Environmental Protection and Biotechnology, Jan Długosz University in Czestochowa, Armii Krajowej 13/15, 42-200 Czestochowa, Poland

**Keywords:** Allotropes, Carbon nanotubes, Fullerene, Graphene, Nanocarbons, Porphyrins

## Abstract

In the review hybrids of cationic porphyrins (*i.e.* porphyrins functionalized by quaternary pyridinium groups) with nanocarbons such as fullerenes, carbon nanotubes and graphene are described. Selected examples of these species are characterized in regard of their properties and possible applications.

## Introduction

*Porphyrins* are planar tetrapyrrolic macrocycles that have interesting properties and value for applications in various fields. They are promising in the design of sensors [1] and they show catalytic properties [2]; some metal porphyrins may be used for analytical purposes [3]. The reactivity of porphyrins enables synthesis of valuable systems, useful in many areas [4–10], *e.g.* design of solar cells [11, 12] and nanowires [13].

Cationic porphyrins, *i.e.* porphyrins meso-functionalized by quaternary azaaromatic units, due to the presence of four positive charges are of interest in the construction of next generation photosensitizers [14]. Cationic porphyrins exist as free ones, and as metalloporphyrins.

The representative compound of cationic porphyrins is meso-tetrakis(*N*-methyl-4-pyridinium porphyrin (TMPyP), in this review referred to as **A**. Among hybrids of cationic porphyrins, those with nanocarbons are a topic of present review.

A growing attention is paid today to interaction of cationic porphyrins with nucleic acids [15]. Cationic porphyrin **A** is a known ^1^O_2_ photosensitizer, effectively used in photodynamic therapy (PDT), generating singlet oxygen for selective destruction of localized tumors [16].

It is noteworthy that antimicrobial photodynamic therapy (aPDT) is now emerging as an alternative to antibiotics; in this procedure the cationic photosensitizers such as cationic porphyrins interact in the excited state with molecular oxygen to produce singlet oxygen that kills the microbial cells [17].

Among a variety of other applications of porphyrins in medicinal field [18] one may mention the use of luminescence of porphyrins for fluorescence diagnostic magnetic resonance imaging [19] and for design of self-illuminating fluorophores suitable for in vivo applications [20].

*Nanocarbons,* belonging to carbon allotropes include zero-dimensional (0D) fullerenes, one-dimensional (1D) carbon nanotubes and two-dimensional (2D) graphene. Nanocarbons have attracted recently a growing attention due to their unique electronic, optical, thermal and chemical properties, promising for their applications in a variety of areas [21].

The smallest species, *i.e.* fullerenes consist of bent sp^2^ carbon atoms. From fullerenes to graphene the strain at sp^2^ carbon atoms decreases; the surfaces of fullerenes and graphene have different reactivities. In fullerenes attempts to release strain by formation of sp^3^ carbon centers exist, therefore fullerenes have anomalously large reactivity, while in graphene no strain at carbon atoms is observed, *i.e.* no tendency for sp^3^ hybridization occurs, and consequently, graphene is less reactive than fullerenes [22].

Nanocarbons are today a topic of an intense research, and a considerable progress in this area is observed. Due to valuable properties they may find applications in various fields; one should point out the importance of these materials so from theoretical and practical viewpoints.

Fullerenes, carbon nanotubes and graphene are of interest in the study of artificial photosynthesis and solar energy conversion, in the construction of electronic, optoelectronic, photovoltaic and sensing devices [23] and in the study of advanced energy conversion (in design of solar cells and fuel cells) and energy storage (in design of batteries and supercapacitors) [24]. Fullerenes and carbon nanotubes are promising as acceptor materials in organic photovoltaics; the donor–acceptor blends with a polymer donor and a fullerene or single walled carbon nanotubes (SWCNT) acceptor were reported [25].

Carbon nanotubes and graphene may be applied in photocatalytic reduction of CO_2_ (produced as an emission from use of hydrocarbon fuels) to hydrocarbon fuels [26]. Carbon nanotubes and graphene are of interest for application in mass and energy transport. They are examples of low dimensional species promising in the reduction of dimensionality of the material [27].

Carbon nanotubes and graphene are investigated in order to facilitate charge transport across abiotic-biotic interfaces [28], they are promising for use in liquid-phase chemiluminescence systems [29] and for design of nano-resonator sensors [30]. The studies of interactions of surfaces of carbon nanotubes and graphene with metal atoms were reported in view of their application in high frequency electronic devices, fuel cells as well as memory and energy devices [31].

Today the use of solar energy is crucial to solve the energy problems for a sustainable society; molecular devices employing hybrids of nanocarbons, (*i.e.* fullerenes, single wall carbon nanotubes SWCNTs and graphene), acting as electron acceptors, with photosensitizers, acting as electron donors, are promising for this aim. In the experiments concerning the above theme, the light-induced electron transfer processes of nanocarbon hybridized with photosensitizers, have been shown to be very useful for this purpose.

Fullerenes act as electron acceptors for electron donors such as cationic porphyrins and phthalocyanines to yield characteristic radical ion pairs, suitable for construction of artificial photosynthetic systems. SWCNTs are also electron acceptors for cationic porphyrins and phthalocyanines. Graphene provides a reaction field of a wide π system for formation of hybrids.

One should emphasize that the electron transfer processes are of a great importance for efficient light-energy harvesting devices such as photovoltaic solar cells [32].

In the paper the noncovalent assemblies of cationic porphyrins with nanocarbons, *i.e.* hybrids of cationic porphyrins with nanocarbons are described in view of their properties and possible applications. The review is a continuation of our former papers concerning cationic porphyrins [33–35], as well as quaternary azaaromatic rotaxanes [36–39] and viologens [40].

## Hybrids of cationic porphyrins with fullerenes

Fullerenes are widely investigated due to their unique structure and interesting properties. They are promising for application in industrial chemistry and electronics; one should also mention construction of polymer/fullerene solar cells. Fullerene derivatives are of interest as antioxidative agents in biological systems. Encapsulation of various species inside fullerenes leads to endohedral fullerenes, valuable in material science and medicine, and in the field of organic photovoltaics. A reflection of the above properties of fullerenes are many reports describing their applications [41–47]. Porphyrins form with fullerenes covalently bound compounds [48–50] or hybrids of porphyrins with fullerenes [51–58], these latter being the theme of the present section.

Hybrids of cationic porphyrins with fullerenes are described below; in these selected examples the dendrofullerenes were used.

### Example 1.1

It was established that octacationic zinc porphyrin ZnP^8+^ forms with anionic C_60_ dendrofullerenes the porph·f and porph_2_·f (porph = ZnP^8+^, f = fullerene) assemblies by electrostatic and charge-transfer interactions; due to these interactions the obtained assemblies are very stable [59, 60]. One should point out that in such assemblies also hydrophobic interaction should be taken into account.

As C_60_ dendrofullerenes the compounds **1–3** have been used. The formation of the assemblies was investigated by gel electrophoresis. For example, the aggregation of ZnP^8+^ with **2** yields the assembly porph·**2,** and with **3** the assemblies porph·**3** and porph_2_·**3** are obtained.

The results of experiments have shown that radical ion pairs ZnP^**·+**^/C_60_^**·−**^ are formed. All radical ion-pair states decay to their singlet ground states without passing through the intermediate triplet excited states of ZnP^8+^ or C_60_ [59, 60].

### Example 1.2

The layer-by-layer (LbL) deposition of positively and negatively charged oligoelectrolyte multilayer (OEM) films was investigated in view of their assembly and disassembly behavior. The LbL technique is a simple and very efficient wet-coating method.

The LbL process involves the alternate deposition of cationic and anionic species from a solution [61–65]. Due to electrostatic attraction and repulsion, the oppositely charged polyions can self-assemble on solid surfaces, *e.g.* on metal or glass. Formation of iterative dipping circles makes it possible to obtain the deposited thin films composed of alternating monolayers of polycations and polyanions. This easy and cheap method affords advanced materials based on polyelectrolyte multilayer films.

The octacationic zinc porphyrin ZnP^8+^ was used as a positively charged oligoelectrolyte, and compounds **4–6** served as negatively charged oligoelectrolytes. By use of electrolytes **5** and **6** containing the porphyrin chromophore, the deposition could be easily followed by UV–Vis spectroscopy [66]. However, using LbL method, polymers serving as charged building blocks are polydisperse and often do not adopt a defined shape within the deposited layers, therefore in experiments the LbL technique was extended to monodisperse oligoelectrolytes with defined structures. It is worth noting that the application of so-called reporter oligoelectrolytes is important, since they enable the precise monitoring of the film assembly by using spectroscopic methods.

Besides the assembly, also the first study of disassembly was made; this investigation is promising in incorporation of bioactive molecules at a given inter-layer position, since after removal of the outer layers the bioactive molecules can be released. Such drug delivery is interesting for biomedical use.

For LbL assembly of molecular oligoelectrolytes, the layers of porphyrin ZnP^8+^ and **4, 5** or **6** were deposited on planar silica substrates using the alternate dipping method. For investigation of possible application of OEMs as drug delivery systems, the biologically active pamidronate **7** used in the bone metastasis was employed as an anionic building block. The porphyrin ZnP^8+^ served as a cationic component and at the same time as a reporter electrolyte, therefore the detection of the anionic component occurred indirectly. It was found that the absorbance of ZnP^8+^ is enhanced when the number of ZnP^8+^ layers increases; the correlation is linear. This behavior enables the regulation of the amount of deposited **7** by the number of layers. 

It was established that the use of chromophore containing electrolytes, in particular porphyrins allows the observation of both assembly and disassembly of OEMs by optical spectroscopy; the compounds containing chromophore serve here as reporter electrolytes. One should point out that oligoelectrolytes used are monodisperse, and various synthetic procedures enable modification of properties of OEMs. It is of interest that the investigated OEMs may show the time dependent release of active components, this behavior being promising for drug delivery [66].

## Hybrids of cationic porphyrins with carbon nanotubes

Carbon nanotubes have interesting mechanical, electronic and optical properties, therefore they are promising for design of composite materials, drug carriers as well as sensing and energy conversion devices. They are investigated with the aim to obtain photo- and redox-responsive materials. Carbon nanotubes, due to their unique structural and electronic properties are of interest for design of hybrids useful in various areas. The applications of nanotubes are today a topic of an enormous amount of reports [67–72].

SWCNTs may be chemically modified for use as nanosized building blocks. This process may be performed by covalent or noncovalent functionalization. The covalent functionalization is rather disadvantageous because of structural changes of nanotubes and loss of some of their unique properties. To overcome this difficulty the noncovalent functionalization of nanotubes is used, this process leads to hybrids of nanotubes with functionalizing agents.

The noncovalent functionalization of nanotubes usually proceeds by π-stacking interactions of an organic molecule or polymer with nanotube walls. It is preferable when the attached molecule has an extended π-electron system of bonds, in this respect the use of porphyrins is convenient. It is noteworthy that in porphyrin/nanotube hybrids, the existence of a light-induced charge transfer between porphyrins and SWCNTs is promising for their application in solar energy conversion.

In the study of mechanism of the hybrid formation it was found that the interaction of nanotubes and porphyrins with charged side residues is much stronger than in the case of neutral porphyrins. Quantum-chemical calculations have shown that the hybrids formed by nanotubes with charged molecules are stabilized due to strong cation-π interactions. It is noteworthy that such complexes are highly stable in water [73]. Hybrids of porphyrins with carbon nanotubes are today intensively studied owing to their promising properties [74–80].

Hybrids of cationic porphyrins with carbon nanotubes are a topic of an intense research, below some examples are presented.

### Example 2.1

It is known that assembling composites of chromophores with carbon nanotubes is a crucial process for construction of photoelectronic devices, biosensors and electron storage devices [81]; for assembling of such composites it is necessary to solubilize nanotubes because they have only low solubility in most solvents. For this purpose the nanostructured LbL ultrathin film consisting of chromophores and single-walled carbon nanotubes (SWNTs) was obtained [62–65, 82]. As chromophores the cationic porphyrin **8** and anionic sodium copper chlorophyllin **9** were used.

The LbL film **8**-SWNT/**9**-SWNT was built from noncovalently adsorbed composites **8-**SWNT and **9**-SWNT. For this purpose SWNTs were dissolved in water soluble cationic **8** and anionic **9**, and the obtained solution served for electrostatic LbL multilayer preparation. The composites were highly dispersed due to existence of π-π interactions [82].

A strong quenching of **8** and **9** fluorescence resulting from their interaction with SWNT was observed. Due to the efficient charge separation and electron transfer in the above LbL film electrodes, the photocurrent generation is enhanced as compared with **8/9** film electrodes without SWNT.

### Example 2.2

In the study of hybrids of porphyrins with carbon nanotubes the interaction of porphyrins A and *meso*-5,10,15,20-tetraphenylporphyrin **10** with zigzag single-walled carbon nanotube (SWNT) has been investigated by resonance Raman spectroscopy and by ab initio and molecular dynamic calculations [73].

The results have shown that the interaction of A with SWNT is stronger than the interaction of **10** with SWNT, since between A and SWNT the cation-π attraction exists. In the molecule A the strong cation-π attraction leads to the saddling; the saddled structure of A provides a closer contact between the charged groups of A with the SWNT surface. The formation of A/SWNT complex in the aqueous solution has been modeled by the molecular dynamics method, showing its stability in the water environment.

### Example 2.3

Photoinduced electron transfer in ion-paired porphyrin/SWNT hybrids has been studied. The donor–acceptor hybrids have been built from water-soluble cationic or anionic porphyrins MA^4+^ or MS^4−^, respectively, serving as electron donors, and from noncovalently functionalized SWNTs serving as electron acceptors. In a first step, SWNTs were solubilized by π-π stacking of pyrene functionalized by anionic (COO^−^) or cationic (NH_3_^+^) groups. Cationic or anionic porphyrins MA^4+^ or MS^4−^ were ion-paired with functionalized SWNT/pyr^−^ and SWNT/pyr^+^ systems to give porphyrin-SWNT donor–acceptor hybrids MA^8+^/SWNT/pyr^−^ or MS^8+^/SWNT/pyr^+^ [83]. The steady-state and time-resolved emission studies have revealed an efficient quenching of the singlet excited state of porphyrins in hybrids. The transient absorption spectra have shown the one-electron oxidation of porphyrins with a simultaneous one-electron reduction of SWNT. 

The charge separation process was further confirmed by an electron mediator (methyl or hexylviologen dication MV^2+^ or HV^2+^, respectively) and an electron–hole shifter 1-benzyl-1,4-dihydronicotinamide (BNAH) in water or DMF, used as solvents. The photoinduced processes resulted in the accumulations of radical cations MV^·+^ and HV^·+^, due to the electron-pooling in the presence of a sacrificial electron donor. This behavior shows the photoinduced electron transfer and enables photocatalytic applications of the above systems.

### Example 2.4

The immobilization of porphyrin A on the carbon nanohorn (CNH) was investigated in order to design donor–acceptor CNH-based hybrids for managing electronic interactions in environmentally friendly aqueous media. In the experiments the water soluble porphyrin A has been immobilized by π-π stacking interactions on carbon nanohorns (CNHs); this process does not disrupt their π-electronic network [84].

The efficient fluorescence quenching of the A unit in the A/CNH assembly suggests charge separations from the photoexcited A to CNH. The photoinduced charge-separation processes within the illuminated A/CNH hybrids, *i.e.* oxidation of the porphyrin and reduction of the nanohorns were studied by transient absorption spectroscopy. In the presence of MV^2+^ and a hole trap, the accumulation of the reduced form of methyl viologen was observed by the illumination of A-CNH; this behavior shows the electron migration from the initially formed charge-separated state [84].

The CNHs were produced by CO_2_ laser ablation of graphite in the absence of metal catalyst under the inert argon atmosphere. The mild sonication of CNH in aqueous solution of A afforded the water soluble A/CNH hybrid. It was found that the morphology of CNH was retained upon immobilization of A, this suggests that the stacking of A onto CNH does not alter the aggregation of CNHs. The high solubilization of CNHs in aqueous solution shows that the surface of CNHs is covered by A.

It is noteworthy that CNHs are more convenient for design of nanomaterials than carbon nanotubes, since they are of a high purity due to the absence of any metal nanoparticles during the laser ablation production, and they have heterogenous surface structure resulting from highly strained conical ends. It is important that the rough surface structure of CNH aggregates enables the better dispersion of CNHs in liquid media than in the case of tightly bundled carbon nanotubes.

In investigation of systems able to mimic natural photosynthesis, the hybrids built from porphyrins and CNHs are of interest. They contain CNHs behaving as electron acceptors and porphyrins as electron donors which act as light-harvesting antennas capturing visible light and transducing the excitation energy. It should be pointed out that the above A/CNH assemblies are promising for use in solar energy conversion.

### Example 2.5

Formation of hybrids of cationic, water soluble metalloporphyrins MP^8+^, namely FeP^8+^ and CoP^8+^ with SWNTs was investigated. For this purpose SWNTs were functionalized with anionic pyrenes from among **11a–d** and treated with metalloporphyrins FeP^8+^ or CoP^8+^. It was observed that the resulting MP^8+^/SWNT/pyr^−^ assemblies form stable hybrid structures in aqueous media (pyr^−^ = pyrene). An important feature of this procedure is that an efficient exfoliation of the initial bundles affords isolated nanohybrid structures [85].

Upon excitation of the above MP^8+^/SWNT/pyr^−^ hybrid with visible light, the rapid intrahybrid electron transfer from the photoexcited MP^8+^ to SWNT occurs. This fact causes the reduction of the electron-accepting SWNT and, at the same time, the oxidation of the electron donating MP^8+^.

One should note the importance of SWNT templates bearing carboxylate or sulfonate groups. These negative groups act as promoters for suspending SWNT samples in aqueous media and as anchors interacting with pyridinium headgroups present in metalloporphyrins. Pyrene compounds are strong fluorophores therefore they are useful sensitive markers in the performed experiments, the fluorescence spectroscopy being a very convenient tool for investigation of the above electron donor–acceptor interactions.

## Hybrids of cationic porphyrins with graphene

Graphene is a single layer of graphite, where carbon atoms are arranged in a honeycomb lattice; owing to its specific electronic and mechanical properties, *e.g.* high charge carrier mobility, graphene is today a topic of an intense research [86–92]. One can say that 2D graphene was wrapped to form 0D fullerenes, was rolled to form 1D carbon nanotubes, and stacked to form 3D graphite [93]. In contrast to fullerenes or nanotubes, graphene is a large anisotropic, very flexible material which may be bent or folded [94].

The selected techniques for preparation of graphene and related species are presented below [95]. The completely insoluble natural graphite powder upon oxidation affords graphite oxide.

*Graphene oxide* (GO) is the single-layered graphite oxide which can be prepared by the exfoliation of graphite oxide. GO having many functional groups such as hydroxyl, aldehyde, carboxyl, epoxy is obtained by exposing graphite powder to strong oxidant solutions, *e.g.* KMnO_4_/H_2_SO_4_ followed by H_2_O_2_ (30 %). Exfoliation is performed by sonication of GO dispersion.

*Chemically converted graphene* (CCG) is prepared by reduction of GO suspensions *e.g.* with hydrazine; in this way a majority of the oxygen functional groups undergoes elimination and the conjugated structure of GO is restored.

*Carboxylic acid modified* GO (CGO) is obtained by sonication of GO under basic conditions, followed by neutralization with HCl; it selectively reserves the carboxylic acid functional groups.

The unique electronic system of graphene results from its high quality 2D crystal lattice in which electrons can move without being scattered off, therefore high electrical conductivity exists. As drawbacks of graphene may be considered its severe stacking as well as its complete insolubility in all solvents including water. Therefore in the investigation of graphene the main topics are its exfoliation from graphite and its solubilization. Chemical methods of exfoliation involve the use of strong acids [96], ionic liquids [97], surfactants [98] or organic solvents [99]; also tip sonication induces graphene exfoliation affording dispersion in organic solvents [100].

In order to improve the chemical compatibility of graphene with diverse media, it is necessary to develop its well-dispersed form. For this purpose covalent and noncovalent functionalization of graphene with various molecules and nanomaterials was investigated in water and in organic solvents [101–103].

Functionalization of graphene offers its new application possibilities. However, the extensive *covalent* functionalization of graphene introduces sp^3^ defects in its lattice; this behavior decreases its high conductivity [104]. To overcome this inconvenience, the *noncovalent* functionalization may be used, in this way the electronic network of graphene is not disrupted [104]. Noncovalent functionalization, as compared with covalent one, preserves the intrinsic properties of graphene and improves its solubilization via hydrophobic interactions and π-π stacking.

In the noncovalent functionalization the planar aromatic organic molecules are employed; they interact through numerous π–π van des Waals forces with the graphitic framework and promote spontaneous exfoliation [105]. For a noncovalent functionalization of graphene (or of GO) the planar aromatic molecules such as porphyrins [106, 107]; phthalocyanines [108] or pyrenes [109] may be used.

Describing graphene it seems noteworthy to mention *graphene*-*coated metal NPs* and *graphenes modified by metal NPs*; four examples concerning this theme will be given (*NP* = nanoparticle).

In the study of *graphene*-*coated metal NPs* two examples will be shown. In the first one the graphene-coated CoNPs **12** which can be functionalized are presented; *e.g.* they can react with 4-nitrobenzene diazonium tetrafluoroborate to give *p*-nitrophenyl derivative **13**, which upon reduction with elemental sulfur affords aminoderivative **14** useful for peptide coupling [21, 110–112]. The mechanism of the graphene-coated CoNPs reaction with aryl diazonium salts involves an electron transfer from graphene to the diazonium ion; upon evolution of nitrogen the reactive phenyl radical is formed which reacts with graphene surface.

In the next example the graphene-coated CoNPs functionalized by (azidomethyl)benzene **15** undergo “click” reaction with stable radical propargyl ether TEMPO **16** yielding **17** which is an efficient catalyst for oxidation of primary and secondary alcohols [113].

In the study of *graphenes modified by NPs*, two examples, *i.e.* graphenes modified by AuNPs and by AgNPs will be shown. Graphene sheets composed from 2 to 10 layers may serve for preparation of graphenes modified by NPs. Chemical exfoliation of graphite in aqueous medium (H_2_SO_4_/KMnO_4_, then 30 % H_2_O_2_) afforded GO; the subsequent thermal treatment of GO (1050 °C, 30 s) promoted further exfoliation.

The oxygen functionalities (hydroxyl and epoxy groups) situated on the graphene surface are reactive sites for chemical modification and for deposition of NPs [114]. The presence of the oxygen functionalities is important for the growth of Au NPs; it was observed that AuNPs cannot deposit on the totally reduced graphene sheets. After reduction with hydrazine, the graphene as an aqueous suspension was treated with HAuCl_4_ solution, and the subsequent reaction with sodium citrate afforded graphene modified by AuNPs. This material may serve as a substrate for surface-enhanced Raman scattering (SERS) [114].

Another method was used for preparation of graphene modified by AgNPs. GO sheets have been anchored onto TiO_2_ films. It was observed that upon UV illumination of TiO_2_/GO films, the photogenerated electrons from TiO_2_ are captured by GO. [115, 116]. These electrons not only reduce GO to RGO, but also become stored across its sp^2^ network. The stored electrons can serve for reduction of metal ions to metal NPs.

In the presence of silver ions, the AgNPs begin deposit onto GO surface opposite TiO_2_, since GO is able to transport electrons through its plane. The above procedure is possible due to the unique property of GO/RGO to shuttle electrons in a direction orthogonal to its plane, and transfer electrons to Ag^+^ ions. The formed semiconductor-graphene-metal (SGM) film is valuable for catalysis and sensor use. These SGM films, tested as SERS sensors produced considerable target molecule signal enhancement, in this way enabling detection of their nanomolar concentrations.

It seems that the above examples concerning graphene-coated NPs and graphenes modified by NPs may be considered as a supplement to the wide field of graphene applications [117–122].

Hybrids of cationic porphyrins with graphene are today intensively studied. Among many works concerning graphenes functionalized by porphyrins [123–126], a special attention has been paid to graphenes noncovalently functionalized by cationic porphyrins, *i.e.* hybrids of cationic porphyrins with graphene [127–131].

### Example 3.1

The noncovalent functionalization of graphene with porphyrins was widely investigated [130, 131]. In order to elucidate this process the interaction of different chemical types of graphene, *i.e*. GO, CCG and CGO with porphyrin A was studied. It was observed that the intermolecular interaction occurs immediately upon mixing graphene with A; in this process A is immobilized on the graphene surface via electrostatic and π-π stacking interactions.

It is worth noting that functional groups on the surface and edge of GO, CCG and CGO play an important role in functionalization of graphenes with A. The strong fluorescence quenching of A upon its interaction with graphene is due to the efficient electron or energy transfer from excited state A to graphene. The red shift of Soret band of A occurring at its interaction with graphene depends on the kind of functional groups of graphene and decreases in the order CCG > CGO > GO [95].

### Example 3.2

In further experiments porphyrin A was integrated via Coulombic interactions to exfoliated graphene, and the possible photoinduced electron/energy transfer phenomena have been investigated [106]. It is known that the wettability of graphene can be tuned as a function of pH from the organic to aqueous phase by addition of amphiphilic block copolymer poly(isoprene b-acrylic acid), further referred to as pol [106]. The stable aqueous dispersions of graphene were prepared using block copolymer pol in the form of its anion at alkaline pH, then the cationic A was integrated to the system taking advantage of opposite charges of graphene dispersions and A^4+^. As a result, the aqueous dispersion of graphene/pol/A^4+^ assembly was formed. It was established by photoluminescence experiments that in this assembly the graphene layers act as electron acceptor, and A is the photoexcited electron donor.

The absorption and fluorescence spectroscopic measurements have shown the presence of electronic communication between A and graphene in the ground and excited states. Basing on experimental results and taking into account the reported redox potential of graphene [132], one may conclude that the quenching of A fluorescence results rather from electron transfer than from energy transfer [106].

### Example 3.3

It is known that the selectivity and improved sensitivity of SERS measurements are desirable for analytical and environmental applications. The RGO-AgNP composite has been studied for improvement of surface-enhanced resonance Raman signal (SERRS) sensitivity of porphyrin A (RGO = reduced GO; NP = nanoparticle). The SERS-active materials include noble metal NPs, such as silver or gold NPs. These NPs provide a localized surface plasmon resonance leading to a large local electromagnetic field which can enhance Raman scattering signals, enabling single molecule SERS [133]. Metal NPs have been combined with graphene to give composites able to SERS sensing [115].

The RGO-AgNP composite is promising for use as a SERS substrate. Graphene has high surface area, therefore is suitable for dispersion of metal NPs. It was observed that metal NPs can be grown directly on graphene by a simple solution-based approach. The intrinsic ability of RGO to adsorb and complex with molecular species facilitates the enhanced SERRS sensing.

The increased sensitivity for SERS detection using RGO-AgNP composite was investigated in terms of RGO-AgNP-target molecule, *i.e*. porphyrin A interaction; in experiments RGO was shown to be an effective substrate for dispersing AgNPs. The red shift of the Soret band of A in UV–Vis absorption spectrophotometry, observed upon complexation of A with RGO-AgNP confirms this behavior [134]. It was established that the use of RGO-AgNP composite results in a significant SERRS enhancement for target molecule A that undergoes complexation with RGO-AgNP.

## Conclusion

Hybrids of cationic porphyrins with nanocarbons, *i.e.* fullerenes, carbon nanotubes and graphene showed to be promising for various applications, therefore recently they are a topic of a wide investigation. In view of the intense progress in the field of porphyrins [135–138], fullerenes [139–142], carbon nanotubes [143–146] and graphene [147–150] one may expect the further development of porphyrin hybrids with nanocarbons which could be used *e.g.* in the area of electronics or photovoltaics.

In a summary it should be pointed out that the theme concerning hybrids of cationic porphyrins with nanocarbons is very large, therefore from among a great number of reports only selected works have been shown in the review. However, one may hope that, although not exhaustive, it would to some extent help to gain better insight into this area, promising for novel, valuable applications.
